# Supplementary material for: Complexome profiling of the Chlamydomonas psb28 mutant reveals TEF5 as an early PSII assembly factor
Source: Plant Cell. 2025 Mar 17;37(6):koaf055. doi: 10.1093/plcell/koaf055 (PMC12164590; doi:10.1093/plcell/koaf055)
Supplement: koaf055_Supplementary_Data [file koaf055_supplementary_data.zip › psb28_interactive.zip!/plots/intensity/qualityPlots/peptides/_Cre03.g208000.t1.1.html]

### Cre03.g208000.t1.1

Trivial name:   
SeqCoverage [median]: 17.90  
Mass: 12.76 kDa  
  
 

| Sequence | MissedCleavage | Mass [Da] | wtIntensity | muIntensity | Score |
| --- | --- | --- | --- | --- | --- |
| SSTVVSLRPDAK | 1 | 1258.69 | 982.44 | 606.47 | 112.75 |
| VLEATTVMTR | 0 | 1119.60 | 434.99 | 683.83 | 79.91 |
|  |  |  | 1417.4 | 1290.3 |  |
